# Prediction of the Benign or Malignant Nature of Pulmonary Pure Ground-Glass Nodules Based on Radiomics Analysis of High-Resolution Computed Tomography Images

**DOI:** 10.3390/tomography10070078

**Published:** 2024-07-05

**Authors:** Xiaoxia Ping, Nan Jiang, Qian Meng, Chunhong Hu

**Affiliations:** Department of Radiology, The First Affiliated Hospital of Soochow University, No. 188, Shizi Street, Suzhou 215006, China; pingxiaoxia@suda.edu.cn (X.P.); jiangnan@suda.edu.cn (N.J.); mengqian1987@suda.edu.cn (Q.M.)

**Keywords:** radiomics, pure ground-glass nodules, malignant, benign

## Abstract

To evaluate the efficacy of radiomics features extracted from preoperative high-resolution computed tomography (HRCT) scans in distinguishing benign and malignant pulmonary pure ground-glass nodules (pGGNs), a retrospective study of 395 patients from 2016 to 2020 was conducted. All nodules were randomly divided into the training and validation sets in the ratio of 7:3. Radiomics features were extracted using MaZda software (version 4.6), and the least absolute shrinkage and selection operator (LASSO) was employed for feature selection. Significant differences were observed in the training set between benign and malignant pGGNs in sex, mean CT value, margin, pleural retraction, tumor–lung interface, and internal vascular change, and then the mean CT value and the morphological features model were constructed. Fourteen radiomics features were selected by LASSO for the radiomics model. The combined model was developed by integrating all selected radiographic and radiomics features using logistic regression. The AUCs in the training set were 0.606 for the mean CT value, 0.718 for morphological features, 0.756 for radiomics features, and 0.808 for the combined model. In the validation set, AUCs were 0.601, 0.692, 0.696, and 0.738, respectively. The decision curves showed that the combined model demonstrated the highest net benefit.

## 1. Introduction

Pulmonary ground-glass nodules (GGNs) are a type of lung lesion that exhibit heightened opacity on computed tomography (CT) scans and do not obscure the underlying bronchial and vascular architectures within the lung parenchyma [1]. Histopathologically, GGNs are caused by partial alveoli filling due to a variety of reasons, such as thickening of the alveolar walls caused by fluid accumulation, cellular infiltration, or fibrosis; partial alveoli collapse; increased capillary blood volume; or a combination of these, which together lead to the partial replacement of lung air [2]. Consequently, GGNs can represent benign lesions such as inflammation, hemorrhage, or localized interstitial fibrosis, as well as lung cancer or pre-cancerous lesions. Some benign GGNs may disappear over time or with anti-inflammatory treatment, while persistent GGNs often indicate a high risk of lung cancer [3]. Succony et al. [4] reported that 37% of GGNs disappear on CT review after three months, whereas 10% of GGNs ultimately develop into invasive lung cancer.

In recent years, with growing health consciousness and the widespread application of high-resolution CT (HRCT) scans of the chest, the detection of lung cancer presenting as GGNs has become increasingly frequent [5]. Currently, the clinical management strategy for pulmonary GGNs, especially pure ground-glass nodules (pGGNs), mainly involves follow-up examinations [6,7]. However, long-term follow-up may impose substantial psychological stress and economic burdens on patients [8,9]. If the benign or malignant nature of GGNs can be predicted at the time of the initial examination, it would significantly reduce the need for unnecessary follow-ups and alleviate concerns about underdiagnosing stable lung cancer nodules. Additionally, it would minimize the resection of benign nodules without delaying lung cancer diagnosis.

Traditional imaging assessment methods, such as evaluating size, morphology, and margin characteristics, are still the primary method for distinguishing benign from malignant lung nodules [3,10]. However, these methods often yield unstable results and lack accuracy in determining the nature of GGNs, particularly for nodules with blurred borders, slow growth, or atypical morphology [11].

In 2012, Lambin et al. [12] introduced the concept of radiomics, revolutionizing the analysis and interpretation of medical images. This field has developed rapidly in recent years. Its applications in pulmonary lesions primarily include predicting the benignity and malignancy of lung lesions [13,14], as well as forecasting the invasiveness of lung cancer [15,16], predicting gene mutations [17,18], and assessing prognosis [19,20]. However, there are fewer studies applying radiomics to the benign and malignant analysis of pGGNs [21].

In this study, we extracted the CT radiomics features of pGGNs, developed a prediction model, and compared it with the radiographic features, aiming to explore the value of radiomics in predicting the malignancy of pGGNs.

## 2. Materials and Methods

### 2.1. Study Population

This study was a retrospective study approved by the Ethics Committee of our hospital, and informed patient consent was waived (approval 2023-No. 203 of the Ethics Committee).

**Figure 1:**
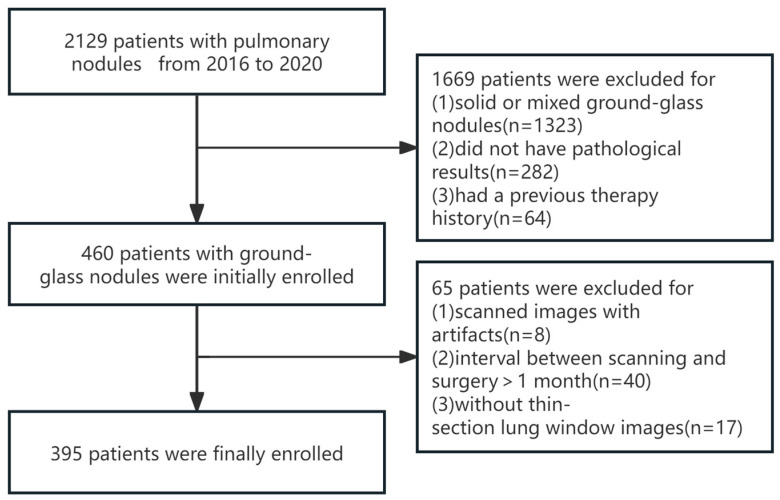
The flow chart shows the inclusion and exclusion criteria of the study.

A comprehensive search was conducted to identify all patients who presented with pulmonary nodules at our institution between 2016 and 2020. Inclusion criteria were as follows: (1) preoperative chest CT scans available in the Picture Archiving and Communication System (PACS) with thin-section lung window images (slice thickness < 1.5 mm); (2) lesions appearing as pure ground-glass nodules; (3) interval between scanning and surgery < 1 month; and (4) complete pathological data. Exclusion criteria were as follows: (1) poor scan quality with significant image artifacts (e.g., respiratory motion artifacts, foreign body artifacts outside the body) not meeting post-processing requirements; (2) invasive diagnostic or therapeutic procedures (e.g., biopsy, radiofrequency ablation) performed before CT scanning; and (3) simultaneously associated with other tumors. Based on the inclusion and exclusion criteria, 395 patients were included in this study, comprising 146 males (51.37 ± 12.14 years) and 249 females (52.45 ± 11.43 years), with 128 benign pGGNs and 267 malignant pGGNs (Figure 1).

All patients were randomly assigned to the training set and validation set in a 7:3 ratio, with 276 cases (89 benign, 187 malignant) in the training set and 119 cases (39 benign, 80 malignant) in the validation set.

### 2.2. Image Acquisition

All patients underwent routine CT scans using multi-slice spiral CT: TOSHIBA Aquilion (Toshiba Medical Systems, Ōtawara, Japan); Somatom Sensation 64, Somatom Definition (Siemens Healthineers, Erlangen, German); GE revolution, Discovery CT 750 HD (GE Healthcare, Chicago, IL, USA). Patients were scanned in the supine position while breath-holding. The scanning range extended from the lung apex to the diaphragm. The scanning parameters were as follows: tube voltage of 100 kV (TOSHIBA), 120 kV (GE, SIMENS), automatic tube current, matrix 512 × 512, and field of view of 400 mm (TOSHIBA), 500 mm (GE, SIMENS). Thin-section lung window images were obtained using standard algorithms with a slice thickness of 1.0 mm or 1.25 mm, window width of 1500 HU, and window level of −600 HU. Subsequently, the acquired images were imported into MaZda software (version 4.6, http://www.eletel.p.lodz.pl/programy/mazda/ (accessed on 11 January 2021)) in a DICOM (digital imaging and communications in medicine) format for analysis.

### 2.3. Image Analysis and Feature Extraction

CT imaging characteristics of pulmonary lesions were evaluated, including mean diameter [(long diameter + short diameter)/2], locate(lobe), CT attenuation value, shape (round/oval, irregular), margin features (spiculated), tumor–lung interface (clear smooth, clear rough, or blurred), pleural retraction (fine linear shadows between the lesion and pleura), vacuole sign (1–3 mm air-containing low-density areas within the lesion), vascular changes including external vascular change (vascular cluster sign) and internal vascular change (thickening, distortion). Measurements and assessments of images were performed by physicians with 5 and 11 years of diagnostic imaging experience, and a consensus was reached through consultation in case of discrepancies.

**Figure 2:**
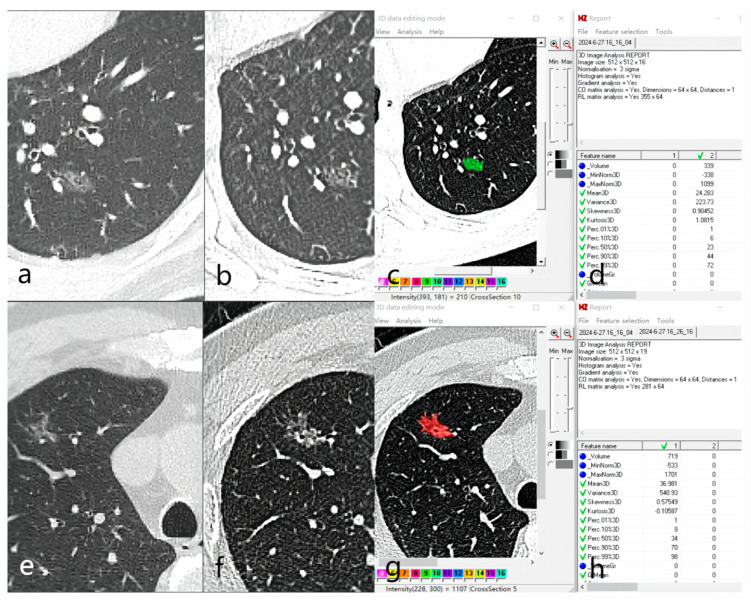
Example of region of interest (ROI) delineation. (**a**–**d**) A 42-year-old male was first discovered with a left lower lobe pure ground-glass nodule (pGGN) in December 2019 (**a**). The follow-up in October 2020 showed no change in the nodule (**b**). (**c**,**d**) show the ROI and the extracted radiomics features. Postoperative pathology revealed focal fibrous tissue proliferation with inflammatory cell infiltration. (**e**–**h**) A 70-year-old male was first discovered with a right upper lobe pGGN in December 2017 (**e**). The follow-up in October 2020 showed an increase in the nodule size (**f**). (**g**,**h**) show the ROI and the extracted radiomics features. Postoperative pathology confirmed adenocarcinoma (papillary + acinar + lepidic subtypes).

After importing the images into the MaZda software, gray-scale normalization was performed using μ ± 3σ to reduce the influence of contrast and brightness variations. The lesion images were then reviewed, and the maximum level of the lesion was selected. The region of interest (ROI) was manually delineated along the lesion contour using the segmentation tool in MaZda software to obtain the radiomics features (Figure 2). The final segmentation of all images was finally completed by the physician with 11 years of experience in obtaining radiomic features.

### 2.4. Statistical Analysis

Graphpad-prism (version Prism 9, https://www.graphpad.com/ (accessed on 15 April 2022)) and R software (version 4.2.2, https://www.r-project.org/ (accessed on 1 February 2023)) were utilized for statistical analysis. Normally distributed continuous data were expressed as mean ± standard deviation (M ± SD) and analyzed using the independent sample *t*-test. Non-normally distributed continuous data were represented as median (interquartile range) [M (Q1, Q3)] and analyzed using the Mann–Whitney U test. Categorical data were presented as numbers (percentages) and analyzed using the chi-square test.

The least absolute shrinkage and selection operator (LASSO) was employed for data dimensionality reduction and feature selection. LASSO is a regularization method for linear regression problems that selects a small number of key features in high-dimensional data, thereby reducing model complexity and preventing overfitting. The selected features were used to construct a predictive model by logistic regression.

The mean CT value, morphological features, radiomics features, and combined model were constructed, and the predictive performance of each model was evaluated using receiver operating characteristic (ROC) curves. A significance level of *p* < 0.05 was considered statistically significant. The clinical net benefit of each predictive model was evaluated using the decision curve analysis (DCA).

## 3. Results

### 3.1. General Information and CT Imaging Features

**Figure 3:**
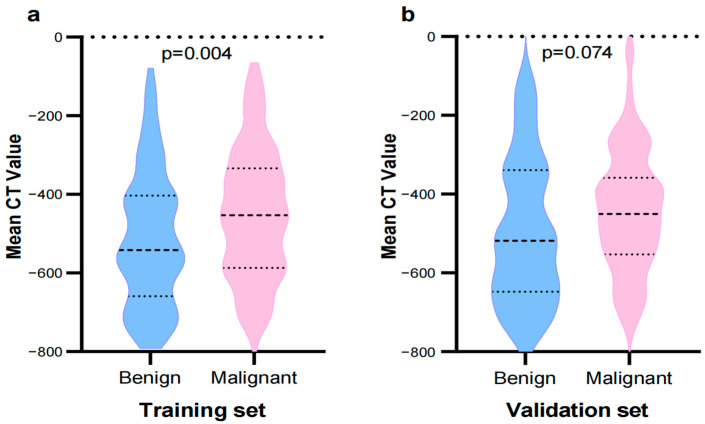
Comparison of the mean CT value for benign pGGNs and malignant pGGNs in the training set (**a**) and the validation set (**b**).

Among the 395 patients, there were 267 cases of pulmonary adenocarcinoma and pre-cancerous lesions, and 128 cases of benign lesions (including chronic inflammation, focal fibrous tissue proliferation, alveolar epithelial hyperplasia, granulomatous inflammation, and carbon deposition). Statistical analysis of clinical data and morphological features of benign pGGNs and malignant pGGNs in the training set revealed significant differences in mean CT value, sex, margin, tumor–lung interface, pleural retraction, internal vascular change (all *p* < 0.05), while age, mean diameter, location, shape, external vascular change, and vacuole sign showed no statistically significant differences (all *p* > 0.05) (Table 1, Figure 3).

### 3.2. Radiomics Feature Analysis

**Figure 4:**
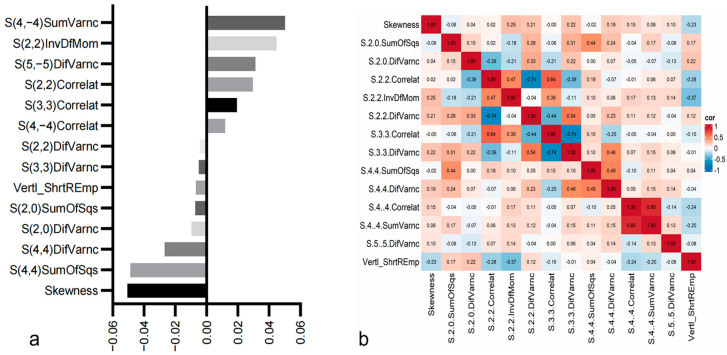
(**a**) Plot of regression coefficients for each feature; (**b**) heat map of correlation coefficients for selected radiomics features.

A total of 304 radiomics features were extracted for each pGGN, including histogram features, absolute gradient features, co-occurrence matrix features, and run-length matrix features [22]. Following LASSO selection, 14 radiomics features associated with the discrimination of benign and malignant lesions were identified, including Skewness, S(2,0)SumOfSqs, S(2,0)DifVarnc, S(2,2)Correlat, S(2,2)InvDfMom, S(2,2)DifVarnc, S(3,3)Correlat, S(3,3)DifVarnc, S(4,4)SumOfSqs, S(4,4)DifVarnc, S(4,−4)Correlat, S(4, −4)SumVarnc, S(5,−5)DifVarnc, Vertl_ShrtREmp. Among these, Skewness represented histogram features, Vertl_ShrtREmp represented gray-level run-length matrix features, and the rest were gray-level co-occurrence matrix features (Figure 4).

### 3.3. Construction and Diagnostic Performance of Predictive Models

Clinical and radiographic features of patients in the training set were analyzed using the chi-square test and Mann–Whitney U test. A model of the mean CT value was developed, along with a predictive model of morphological features (multivariate logistic regression was performed by fitting statistically significant characteristics, except the mean CT value). The area under the curve (AUC) of the mean CT value model in the training set was 0.606 [95% confidence interval (CI) 0.534–0.678], and that of the morphological prediction model was 0.718 (95% CI 0.656–0.781). In the validation set, the AUC of the mean CT value model was 0.601 (95% CI 0.486–0.717), and that of the morphological model was 0.692 (95% CI 0.589–0.795).

**Figure 5:**
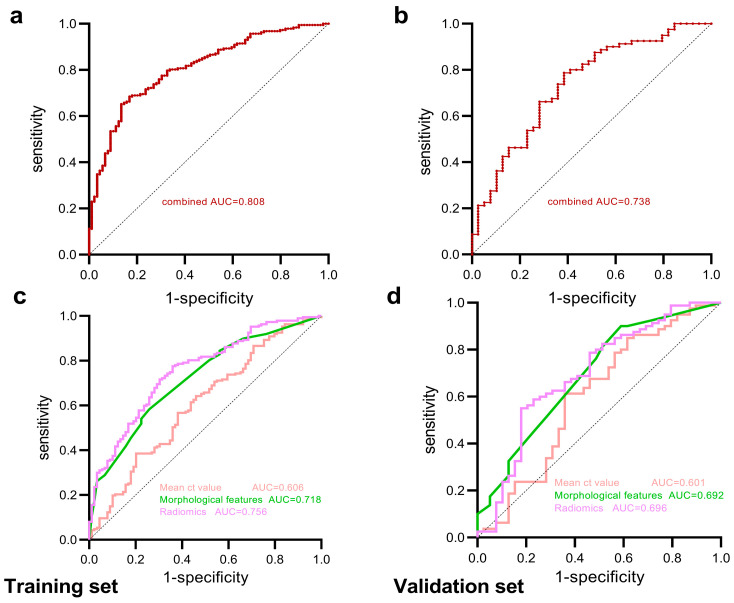
ROC curves of the combined model, mean CT value model, morphological features model, and radiomics model in the training set (**a**,**c**) and the validation set (**b**,**d**).

**Figure 6:**
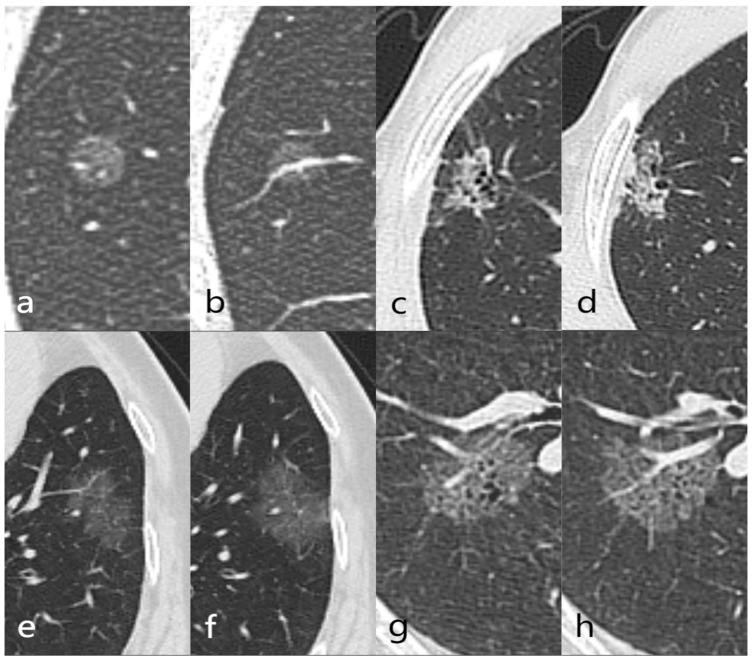
Examples of the combined predictive model successfully and unsuccessfully classifying the studied nodules. (**a**,**b**) A 60-year-old male with a pure ground-glass nodule (pGGN) in the right lower lobe. The nodule is round-shaped with a clear-smooth tumor–lung interface and no spiculation. Postoperative pathology revealed fibrous tissue hyperplasia with inflammatory cell infiltration. The predictive model classified the nodule as benign. (**c**,**d**) A 52-year-old male with a pGGN in the right upper lobe. The nodule is irregularly shaped with a clear-rough tumor–lung interface, spiculation, pleural retraction, vacuole sign, and vessel convergence sign. Postoperative pathology revealed chronic inflammation with stromal fibrous tissue hyperplasia and glandular hyperplasia. The predictive model classified the nodule as malignant. (**e**,**f**) A 67-year-old female with a pGGN in the left lower lobe. The nodule is irregularly shaped with spiculation and internal vascular distortion. Postoperative pathology revealed adenocarcinoma (acinar + lepidic subtypes). The predictive model classified the nodule as malignant. (**g**,**h**) A 59-year-old male with a pGGN in the right lower lobe. The nodule is round-shaped with a vacuole sign and a normal vascular course within the lesion. Postoperative pathology revealed adenocarcinoma (lepidic + acinar subtypes). The predictive model classified the nodule as benign.

**Figure 7:**
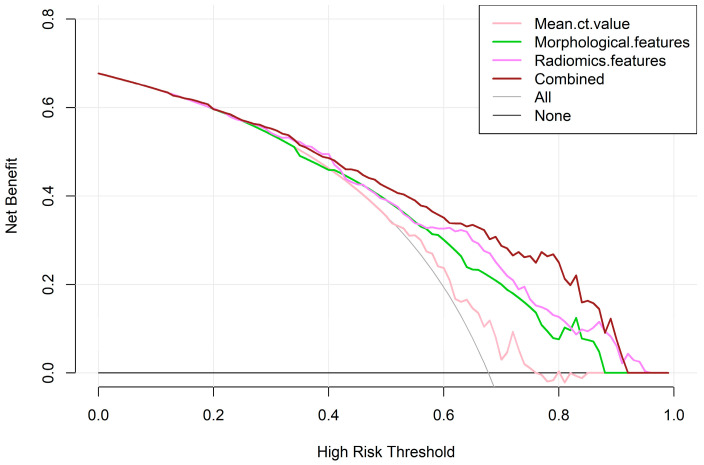
The decision curve analysis showed that the net benefit of the combined model was greater than that of the other three predictive models.

A radiomics predictive model was constructed based on the 14 features selected by LASSO. The AUC was 0.756 (95% CI 0.696–0.815) in the training set and 0.696 (95% CI 0.590–0.802) in the validation set. The mean CT value, selected morphological features, and radiomics features were fitted by logistic regression, and a combined model was constructed in an AUC of 0.808 (95% CI 0.755–0.861) in the training set and 0.738 (95% CI 0.641–0.835) in the validation set (Table 2, Figure 5). The mean CT value model had a lower predictive performance, the radiomics model outperformed the morphological features model, and the combined model had the highest predictive performance (Delong test: *p* < 0.05) (Table 3). Figure 6 illustrates examples of the combined model successfully or unsuccessfully classifying the studied nodules. Meanwhile, the DCA showed that the clinical net benefit of the combined model was greater than that of the other three predictive models (Figure 7).

## 4. Discussion

Globally, approximately 1.8 million people die from lung cancer each year, making it the leading cause of cancer-related deaths [23]. Furthermore, lung cancer accounts for the highest number of disability-adjusted life years across all age groups of cancer patients, irrespective of gender [24], indicating that lung cancer results in the greatest loss of healthy life years. About 75% of patients are already in advanced stages at the time of diagnosis, and the overall 5-year survival rate for patients with advanced lung cancer is only about 20% [25]. In contrast, patients with adenocarcinoma in situ and microinvasive adenocarcinoma who undergo early-stage, complete surgical resection have a 10-year disease-specific survival rate of 100% [26]. This significant disparity indicates that the key to improving lung cancer survival rates lies in early detection, diagnosis, and timely surgical intervention.

Previous studies have shown that the CT value of GGNs can be used to distinguish between benign and malignant lesions and to differentiate the invasiveness of lung adenocarcinoma [3,27,28]. Yang et al. [3] suggested that a higher mean CT value in pGGNs may be advantageous for diagnosing malignant tumors, with values of −550 ± 141 HU for malignant lesions compared to −645 ± 90 HU for benign ones (*p* < 0.05). Wang et al. [27] conducted an analysis of thin-section CT images of 154 cases with sub-solid nodules and found that pre-invasive and micro-invasive lesions had lower CT value (−396.81 ± 235.20 HU) than invasive lesions (−191.64 ± 206.23 HU, *p* < 0.001). In this study, a statistically significant difference was observed in the mean CT value between benign and malignant pGGNs (−541.56 HU vs. −452.9 HU, *p* = 0.004). Then, constructing an ROC curve using mean CT value, the AUC for the training set was 0.606 (95% CI 0.534–0.678), and for the validation set, it was 0.601 (95% CI 0.486–0.717). This suggests that although there is a difference in mean CT value between benign and malignant pGGNs, and the diagnostic performance assessed by CT values alone is limited.

Traditional CT imaging feature analysis can also be used to diagnose the benignity or malignancy of lung nodules [3,10,29]. Yang et al. [3] performed a multivariate analysis in the pGGNs subgroup, and discovered that a well-defined border was a significant predictor favoring the diagnosis of malignancy; the AUC for this predictor was 0.705 (95% CI 0.583–0.828). In this study, there are four significant differences observed in HRCT imaging manifestations of pGGNs, including margin, pleural retraction, tumor–lung interface, and internal vascular change, between benign and malignant cases (all *p* < 0.05). It indicates that certain morphological features like margin spiculation, pleural retraction, and vascular changes within the lesions were more common in the malignant pGGNs compared to the benign ones. These may attributed to the rapid growth of adenocarcinoma and the irregular rate of internal growth of the lesion, resulting in uneven infiltration of the surrounding structures [10,30,31]. Using logistic regression, a morphological feature model was established, with an AUC of 0.718 (95% CI 0.656–0.781) in the training set and an AUC of 0.692 (95% CI 0.589–0.795) in the validation set, demonstrating moderate diagnostic performance. Although morphological analysis and interpretation of CT images contribute to disease diagnosis, these rely on the expertise and understanding of imaging manifestations by diagnostic physicians. Moreover, when lesions present as pGGNs, the specificity of imaging features is insufficient, which can affect the accuracy of image interpretation.

Radiomics, by extracting and analyzing a large number of quantitative imaging features from medical images, can capture subtle differences in tissue characteristics that may not be discernible by the naked eye alone. While benign and malignant lesions may both manifest as pure ground-glass opacities on HRCT scans, the inherent pathophysiological characteristics and high degree of histological heterogeneity of lung cancer significantly distinguish it from benign lesions. Radiomics analysis can extract and quantify these intrinsic variations within tissue structures, and offer a nuanced perspective that transcends conventional imaging assessments [32,33]. Gong et al. [33] employed radiomics analysis to diagnose ground-glass opacities in four datasets, achieving AUC values of 0.75, 0.55, 0.77, and 0.93. The accuracy was higher than that of two radiologists (53.1%, 56.3%, respectively). In this study, a total of 14 radiomics features with high diagnostic value were selected to establish the predictive model, with an AUC of 0.756 (95% CI 0.696–0.815) in the training set and an AUC of 0.696 (95% CI 0.590–0.802) in the validation set. The predictive value of the radiomics model was higher than the mean CT value and the morphological features model.

The radiomics model demonstrates strong performance in capturing microstructural variations in lesions, while clinical and radiographic features primarily reflect macroscopic manifestations and patients’ background information. The integration of both approaches (e.g., the combined model) theoretically allows for a more comprehensive assessment of the pGGNs. In the study, we constructed the combined model and compared it to the radiomics, the mean CT value, and the morphological model. The combined model exhibited the highest predictive value in both the training set (AUC = 0.808, 95% CI 0.755–0.861) and the validation set (AUC = 0.738, 95% CI 0.641–0.835) (Delong test, *p* < 0.05). Compared to individual models, the combined model demonstrated superior diagnostic efficacy, highlighting the potential of radiomics in enhancing diagnostic precision.

This study also has some limitations. Firstly, this is a single-center retrospective study, which raises the possibility of data bias. Secondly, there is inconsistency among the CT scanners used for the cases included in this study, potentially impacting the results despite image normalization efforts. To address these limitations and provide more robust validation of our findings, future studies will involve collaborative multicenter efforts, which would help reduce data bias by incorporating a more diverse patient population and a wider range of imaging equipment. Additionally, multiparameter studies that incorporate various imaging modalities and clinical parameters could offer a more comprehensive assessment and strengthen the reliability of the predictive models. 

## 5. Conclusions

In conclusion, we analyzed the clinical data, morphological features, and radiomics features of pGGNs, and developed a combined model that can non-invasively predict the benign or malignant nature of pGGNs. This model has the potential to significantly aid in clinical diagnosis and decision-making processes.

## Figures and Tables

**Table 1:** Clinical and morphological features of benign and malignant pure ground-glass nodules in the training set.

Variables	Total (n = 276)	Benign (n = 89)	Malignant (n = 187)	Statistic	*p*
Age, mean ± SD	52.42 ± 11.88	51.54 ± 12.30	52.83 ± 11.68	t = −0.85	0.398
Mean diameter, M (Q_1_, Q_3_)	10.03 (7.68, 13.34)	9.31 (7.67, 12.59)	10.36 (7.72, 14.07)	Z = −1.53	0.127
Mean CT value, M (Q_1_, Q_3_)	−477.30 (−599.59, −356.35)	−541.56 (−647.60, −403.13)	−452.90 (−585.46, −337.68)	Z = −2.85	0.004
Sex, n (%)				χ^2^ = 15.62	<0.001
female	176 (63.77)	42 (47.19)	134 (71.66)		
male	100 (36.23)	47 (52.81)	53 (28.34)		
Location, n (%)				χ^2^ = 5.50	0.24
RUL	96 (34.78)	34 (38.20)	62 (33.16)		
RML	23 (8.33)	3 (3.37)	20 (10.70)		
RLL	43 (15.58)	17 (19.10)	26 (13.90)		
LUL	71 (25.72)	22 (24.72)	49 (26.20)		
LLL	43 (15.58)	13 (14.61)	30 (16.04)		
Shape, n (%)				χ^2^ = 0.50	0.481
round or oval	197 (71.38)	66 (74.16)	131 (70.05)		
irregular	79 (28.62)	23 (25.84)	56 (29.95)		
Margin, n (%)				χ^2^ = 4.63	0.031
not spiculated	180 (65.22)	66 (74.16)	114 (60.96)		
spiculated	96 (34.78)	23 (25.84)	73 (39.04)		
Tumor−lung interface, n (%)				χ^2^ = 14.82	<0.001
clear smooth	130 (47.10)	48 (53.93)	82 (43.85)		
clear rough	78 (28.26)	12 (13.48)	66 (35.29)		
blurry	68 (24.64)	29 (32.58)	39 (20.86)		
Pleural, n (%)				χ^2^ = 11.54	<0.001
no retraction	199 (72.10)	76 (85.39)	123 (65.78)		
retraction	77 (27.90)	13 (14.61)	64 (34.22)		
External vascular change, n (%)				χ^2^ = 2.66	0.103
absence	102 (36.96)	39 (43.82)	63 (33.69)		
presence	174 (63.04)	50 (56.18)	124 (66.31)		
Internal vascular change, n (%)				χ^2^ = 14.10	<0.001
absence	144 (52.17)	61 (68.54)	83 (44.39)		
presence	132 (47.83)	28 (31.46)	104 (55.61)		
Vacuole sign, n (%)				χ^2^ = 1.90	0.168
absence	226 (81.88)	77 (86.52)	149 (79.68)		
presence	50 (18.12)	12 (13.48)	38 (20.32)		

t: *t*-test, Z: Mann–Whitney test, χ^2^: chi-square test, SD: standard deviation, M: median, Q_1_: first quartile, Q_3_: third quartile, RUL: right upper lobe, RML: right middle lobe, RLL: right lower lobe, LUL: left upper lobe, LLL: left lower lobe.

**Table 2:** Predictive performance of the four models in the training and validation sets.

	Models	Area	95% CI	Specificity	Sensitivity	NPV	PPV	FDR	FPR
Training set	Mean CT value	0.606	0.534–0.678	0.618	0.567	0.404	0.757	0.243	0.382
	Morphological features	0.718	0.656–0.781	0.742	0.583	0.458	0.826	0.174	0.258
	Radiomics features	0.756	0.696–0.815	0.640	0.775	0.576	0.819	0.181	0.360
	Combined	0.808	0.755–0.861	0.865	0.652	0.542	0.910	0.090	0.135
Validation set	Mean CT value	0.601	0.486–0.717	0.641	0.613	0.446	0.778	0.222	0.359
	Morphological features	0.692	0.589–0.795	0.410	0.900	0.667	0.758	0.242	0.590
	Radiomics features	0.696	0.590–0.802	0.821	0.550	0.471	0.863	0.137	0.179
	Combined	0.738	0.641–0.835	0.615	0.788	0.585	0.808	0.192	0.385

CI: confidence interval, PPV: positive predictive value, NPV: negative predictive value, FDR: false discovery rate, FPR: false position rate.

**Table 3:** The Delong test between the prediction models in the training set.

Models	Statistic	*p*	95% Confidence Interval
Combined vs. Radiomics	2.509	0.012	0.011 to 0.093
Combined vs. Morphological	3.500	<0.001	0.039 to 0.139
Combined vs. Mean CT value	5.016	<0.001	0.123 to 0.281
Radiomics vs. Morphological	0.881	0.378	−0.046 to 0.119
Radiomics vs. Mean CT value	3.561	<0.001	0.067 to 0.232
Morphological vs. Mean CT value	2.548	0.011	0.026 to 0.199

## Data Availability

The datasets used during the current study were available from the corresponding author on reasonable request.

## References

[1] Austin J.H., Müller N.L., Friedman P.J., Hansell D.M., Naidich D.P., Remy-Jardin M., Webb W.R., Zerhouni E.A. (1996). Glossary of Terms for CT of the Lungs: Recommendations of the Nomenclature Committee of the Fleischner Society. Radiology.

[2] Lee H.Y., Choi Y.-L., Lee K.S., Han J., Zo J.I., Shim Y.M., Moon J.W. (2014). Pure Ground-Glass Opacity Neoplastic Lung Nodules: Histopathology, Imaging, and Management. Am. J. Roentgenol..

[3] Yang W., Sun Y., Fang W., Qian F., Ye J., Chen Q., Jiang Y., Yu K., Han B. (2018). High-Resolution Computed Tomography Features Distinguishing Benign and Malignant Lesions Manifesting as Persistent Solitary Subsolid Nodules. Clin. Lung Cancer.

[4] Succony L., Rassl D.M., Barker A.P., McCaughan F.M., Rintoul R.C. (2021). Adenocarcinoma Spectrum Lesions of the Lung: Detection, Pathology and Treatment Strategies. Cancer Treat. Rev..

[5] McWilliams A., Tammemagi M.C., Mayo J.R., Roberts H., Liu G., Soghrati K., Yasufuku K., Martel S., Laberge F., Gingras M. (2013). Probability of Cancer in Pulmonary Nodules Detected on First Screening CT. N. Engl. J. Med..

[6] MacMahon H., Naidich D.P., Goo J.M., Lee K.S., Leung A.N.C., Mayo J.R., Mehta A.C., Ohno Y., Powell C.A., Prokop M. (2017). Guidelines for Management of Incidental Pulmonary Nodules Detected on CT Images: From the Fleischner Society 2017. Radiology.

[7] Wood D.E., Kazerooni E.A., Baum S.L., Eapen G.A., Ettinger D.S., Hou L., Jackman D.M., Klippenstein D., Kumar R., Lackner R.P. (2018). Lung Cancer Screening, Version 3.2018, NCCN Clinical Practice Guidelines in Oncology. J. Natl. Compr. Cancer Netw..

[8] Lee H.W., Jin K.-N., Lee J.-K., Kim D.K., Chung H.S., Heo E.Y., Choi S.H. (2019). Long-Term Follow-Up of Ground-Glass Nodules After 5 Years of Stability. J. Thorac. Oncol..

[9] Kobayashi Y., Fukui T., Ito S., Usami N., Hatooka S., Yatabe Y., Mitsudomi T. (2013). How Long Should Small Lung Lesions of Ground-Glass Opacity Be Followed?. J. Thorac. Oncol..

[10] Liang Z.-R., Ye M., Lv F.-J., Fu B.-J., Lin R.-Y., Li W.-J., Chu Z.-G. (2022). Differential Diagnosis of Benign and Malignant Patchy Ground-Glass Opacity by Thin-Section Computed Tomography. BMC Cancer.

[11] Liu A., Wang Z., Yang Y., Wang J., Dai X., Wang L., Lu Y., Xue F. (2020). Preoperative Diagnosis of Malignant Pulmonary Nodules in Lung Cancer Screening with a Radiomics Nomogram. Cancer Commun..

[12] Lambin P., Rios-Velazquez E., Leijenaar R., Carvalho S., van Stiphout R.G.P.M., Granton P., Zegers C.M.L., Gillies R., Boellard R., Dekker A. (2012). Radiomics: Extracting More Information from Medical Images Using Advanced Feature Analysis. Eur. J. Cancer.

[13] Zhang T., Yuan M., Zhong Y., Zhang Y.-D., Li H., Wu J.-F., Yu T.-F. (2019). Differentiation of Focal Organising Pneumonia and Peripheral Adenocarcinoma in Solid Lung Lesions Using Thin-Section CT-Based Radiomics. Clin. Radiol..

[14] Elia S., Pompeo E., Santone A., Rigoli R., Chiocchi M., Patirelis A., Mercaldo F., Mancuso L., Brunese L. (2023). Radiomics and Artificial Intelligence Can Predict Malignancy of Solitary Pulmonary Nodules in the Elderly. Diagnostics.

[15] Yang Y., Tan M., Ma W., Duan S., Huang X., Jin L., Tang L., Li M. (2022). Preoperative Prediction of the Degree of Differentiation of Lung Adenocarcinoma Presenting as Sub-Solid or Solid Nodules with a Radiomics Nomogram. Clin. Radiol..

[16] Zhang C.-R., Wang Q., Feng H., Cui Y.-Z., Yu X.-B., Shi G.-F. (2023). Computed-Tomography-Based Radiomic Nomogram for Predicting the Risk of Indeterminate Small (5–20 Mm) Solid Pulmonary Nodules. Diagn. Interv. Radiol..

[17] He R., Yang X., Li T., He Y., Xie X., Chen Q., Zhang Z., Cheng T. (2022). A Machine Learning-Based Predictive Model of Epidermal Growth Factor Mutations in Lung Adenocarcinomas. Cancers.

[18] Sacconi B., Anzidei M., Leonardi A., Boni F., Saba L., Scipione R., Anile M., Rengo M., Longo F., Bezzi M. (2017). Analysis of CT Features and Quantitative Texture Analysis in Patients with Lung Adenocarcinoma: A Correlation with EGFR Mutations and Survival Rates. Clin. Radiol..

[19] Zhang R., Wei Y., Shi F., Ren J., Zhou Q., Li W., Chen B. (2022). The Diagnostic and Prognostic Value of Radiomics and Deep Learning Technologies for Patients with Solid Pulmonary Nodules in Chest CT Images. BMC Cancer.

[20] Sun Q., Huang Y., Wang J., Zhao S., Zhang L., Tang W., Wu N. (2019). Applying CT Texture Analysis to Determine the Prognostic Value of Subsolid Nodules Detected during Low-Dose CT Screening. Clin. Radiol..

[21] Kao T.-N., Hsieh M.-S., Chen L.-W., Yang C.-F.J., Chuang C.-C., Chiang X.-H., Chen Y.-C., Lee Y.-H., Hsu H.-H., Chen C.-M. (2022). CT-Based Radiomic Analysis for Preoperative Prediction of Tumor Invasiveness in Lung Adenocarcinoma Presenting as Pure Ground-Glass Nodule. Cancers.

[22] Strzelecki M., Szczypinski P., Materka A., Klepaczko A. (2013). A Software Tool for Automatic Classification and Segmentation of 2D/3D Medical Images. Nucl. Instrum. Methods Phys. Res. Sect. A Accel. Spectrometers Detect. Assoc. Equip..

[23] Sung H., Ferlay J., Siegel R.L., Laversanne M., Soerjomataram I., Jemal A., Bray F. (2021). Global Cancer Statistics 2020: GLOBOCAN Estimates of Incidence and Mortality Worldwide for 36 Cancers in 185 Countries. CA A Cancer J. Clin..

[24] Qiu H., Cao S., Xu R. (2021). Cancer Incidence, Mortality, and Burden in China: A Time-Trend Analysis and Comparison with the United States and United Kingdom Based on the Global Epidemiological Data Released in 2020. Cancer Commun..

[25] Allemani C., Matsuda T., Di Carlo V., Harewood R., Matz M., Nikšić M., Bonaventure A., Valkov M., Johnson C.J., Estève J. (2018). Global Surveillance of Trends in Cancer Survival 2000-14 (CONCORD-3): Analysis of Individual Records for 37 513 025 Patients Diagnosed with One of 18 Cancers from 322 Population-Based Registries in 71 Countries. Lancet.

[26] Nakada T., Kuroda H. (2021). Narrative Review of Optimal Prognostic Radiological Tools Using Computed Tomography for T1N0-Staged Non-Small Cell Lung Cancer. J. Thorac. Dis..

[27] Wang H., Weng Q., Hui J., Fang S., Wu X., Mao W., Chen M., Zheng L., Wang Z., Zhao Z. (2020). Value of TSCT Features for Differentiating Preinvasive and Minimally Invasive Adenocarcinoma From Invasive Adenocarcinoma Presenting as Subsolid Nodules Smaller Than 3 Cm. Acad Radiol..

[28] Li Q., Fan L., Cao E.-T., Li Q.-C., Gu Y.-F., Liu S.Y. (2017). Quantitative CT Analysis of Pulmonary Pure Ground-Glass Nodule Predicts Histological Invasiveness. Eur. J. Radiol..

[29] Bianconi F., Palumbo I., Fravolini M.L., Rondini M., Minestrini M., Pascoletti G., Nuvoli S., Spanu A., Scialpi M., Aristei C. (2022). Form Factors as Potential Imaging Biomarkers to Differentiate Benign vs. Malignant Lung Lesions on CT Scans. Sensors.

[30] Yang Y., Li K., Sun D., Yu J., Cai Z., Cao Y., Wu J. (2019). Invasive Pulmonary Adenocarcinomas Versus Preinvasive Lesions Appearing as Pure Ground-Glass Nodules: Differentiation Using Enhanced Dual-Source Dual-Energy CT. AJR Am. J. Roentgenol..

[31] Gao F., Li M., Zhang Z., Xiao L., Zhang G., Zheng X., Hua Y., Li J. (2019). Morphological Classification of Pre-Invasive Lesions and Early-Stage Lung Adenocarcinoma Based on CT Images. Eur. Radiol..

[32] Liang G., Yu W., Liu S.-Q., Xie M.-G., Liu M. (2022). The Value of Radiomics Based on Dual-Energy CT for Differentiating Benign from Malignant Solitary Pulmonary Nodules. BMC Med. Imaging.

[33] Gong J., Liu J., Hao W., Nie S., Wang S., Peng W. (2019). Computer-Aided Diagnosis of Ground-Glass Opacity Pulmonary Nodules Using Radiomic Features Analysis. Phys. Med. Biol..

